# Posterior Tibial Nerve Schwannoma Presenting as Tarsal Tunnel Syndrome

**DOI:** 10.7759/cureus.5303

**Published:** 2019-08-01

**Authors:** Aaradhana J Jha, Chandan R Basetty, Gean C Viner, Chandler Tedder, Ashish Shah

**Affiliations:** 1 Orthopaedics, University of Alabama School of Medicine, Birmingham, USA; 2 Orthopaedics, University of South Alabama, Mobile, USA

**Keywords:** schwannoma, tarsal tunnel syndrome, posterior tibial nerve

## Abstract

Schwannomas are rare, benign tumors originating in the Schwann cells of the peripheral nervous system. They are most commonly found in the head, neck, and upper extremities, which involve the spinal nerves of the brachial plexus. However, schwannomas of the lower extremities are extremely uncommon, and few studies have reported a schwannoma originating from the posterior tibial nerve. We report on a case of a 71-year old male who presented to our clinic because of left foot and ankle neuritic pain. A nerve tumor was found; subsequently, the tumor was surgically excised along with the release of the tarsal tunnel.

## Introduction

A schwannoma is a benign nerve sheath tumor arising from the Schwann cells of the peripheral nervous system. They tend to be isolated, slow-growing, and well-encapsulated neoplasms that form within the perineurium [1-5]. An exception to this is when they are associated with neurofibromatosis. Although some reports have suggested an association with prior trauma [3,4], schwannomas commonly present in the fourth decade of life with no gender bias reported in the literature and an infrequent rate of malignant transformation [1,2,4-6].

Schwannomas most commonly occur in the head and neck region involving the spinal nerves and the brachial plexus. They are a rare occurrence in the lower extremities, particularly in the foot and ankle, with the most common nerve affected being the posterior tibial nerve [2-5,7-9]. Solitary schwannomas can present as an asymptomatic lump or cause compressive neuropathy due to a mass effect and displacement of nerve bundles. A typical presentation is pain and numbness of the plantar foot which can be mistaken to be due to lumbar radiculopathy. Diagnosis can be missed or delayed due to the slow growth of these tumors, leading to the development of tarsal tunnel syndrome because of compression of the posterior tibial nerve [4,7-13]. Schwannomas can be carefully resected without damage to the adjacent nerve due to their nerve sheath origin [2,5].

We report a case of a patient with a lower extremity schwannoma affecting the posterior tibial nerve with symptoms of tarsal tunnel syndrome.

## Case presentation

A 71-year-old male with no significant past medical history presented to our clinic with left foot and ankle pain for six years. His pain had been increasing progressively in intensity as well as frequency. He described the pain as electric and burning in nature, located in the plantar aspect of his foot and radiating to the arch of the foot along the first two toes. He had been diagnosed with Morton’s neuroma, tarsal tunnel syndrome and peripheral neuropathy at an outside hospital for which he had been treated with narcotics and gabapentin. After a year of treatment with conservative measures, he failed to respond, and MRI was performed which revealed a nerve tumor. He presented to our center seeking options for removal of the tumor.

Apart from obesity, hypertension and coronary arteriosclerosis, he did not have any co-morbid conditions. He did not have any significant family or allergy history. He smoked more than 10 cigarettes per day.

A review of systems was negative. Examination of his left leg, foot and ankle indicated that he was tender to palpation in the calf along the posteromedial and supramalleolar regions. These regions had a positive Tinel’s sign with radiation to the arch of foot along calcaneal, medial and lateral branches of the tibial nerve. All other neurovascular signs were normal.

**Figure 1:**
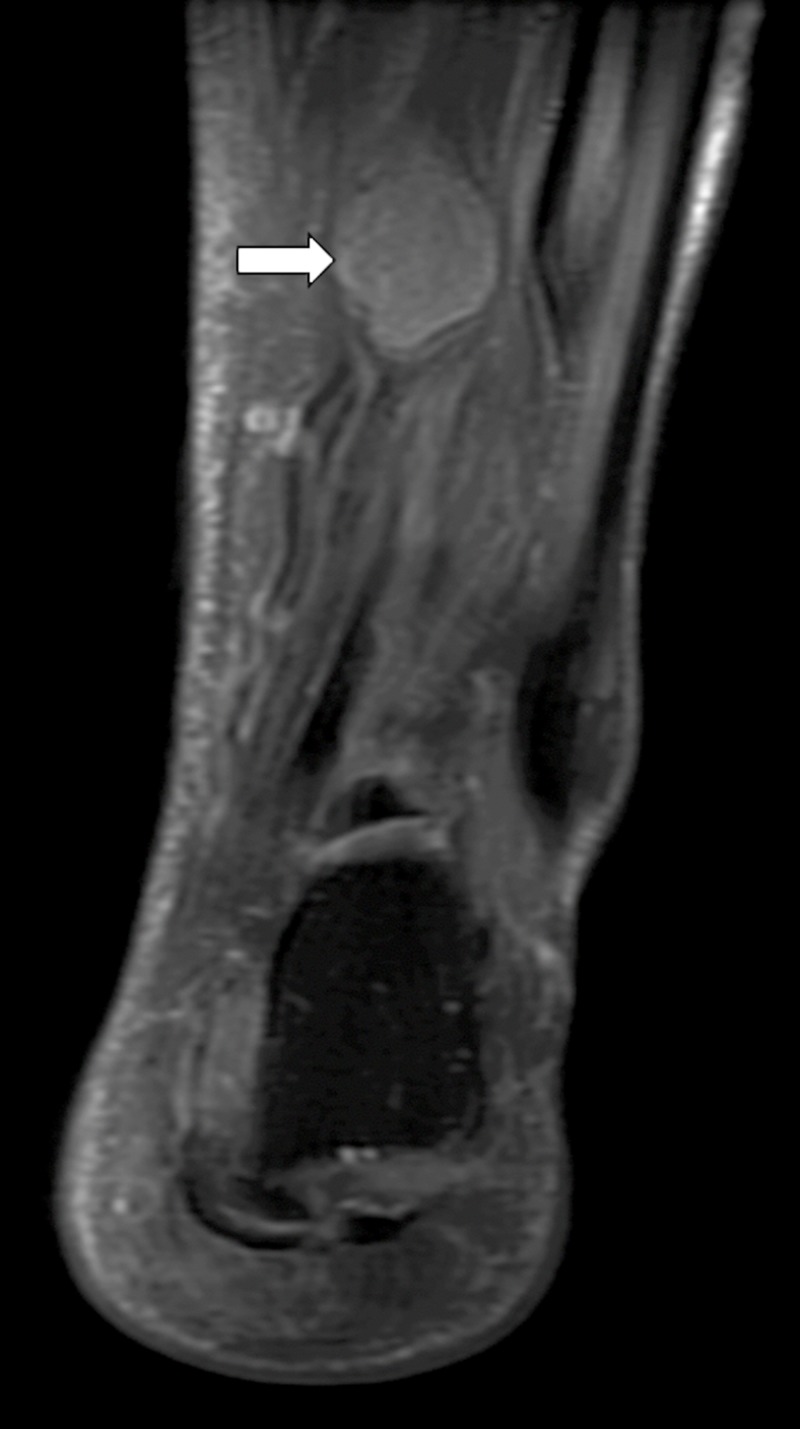
T2 weighted coronal MRI showing the mass arising from posterior tibial nerve

**Figure 2:**
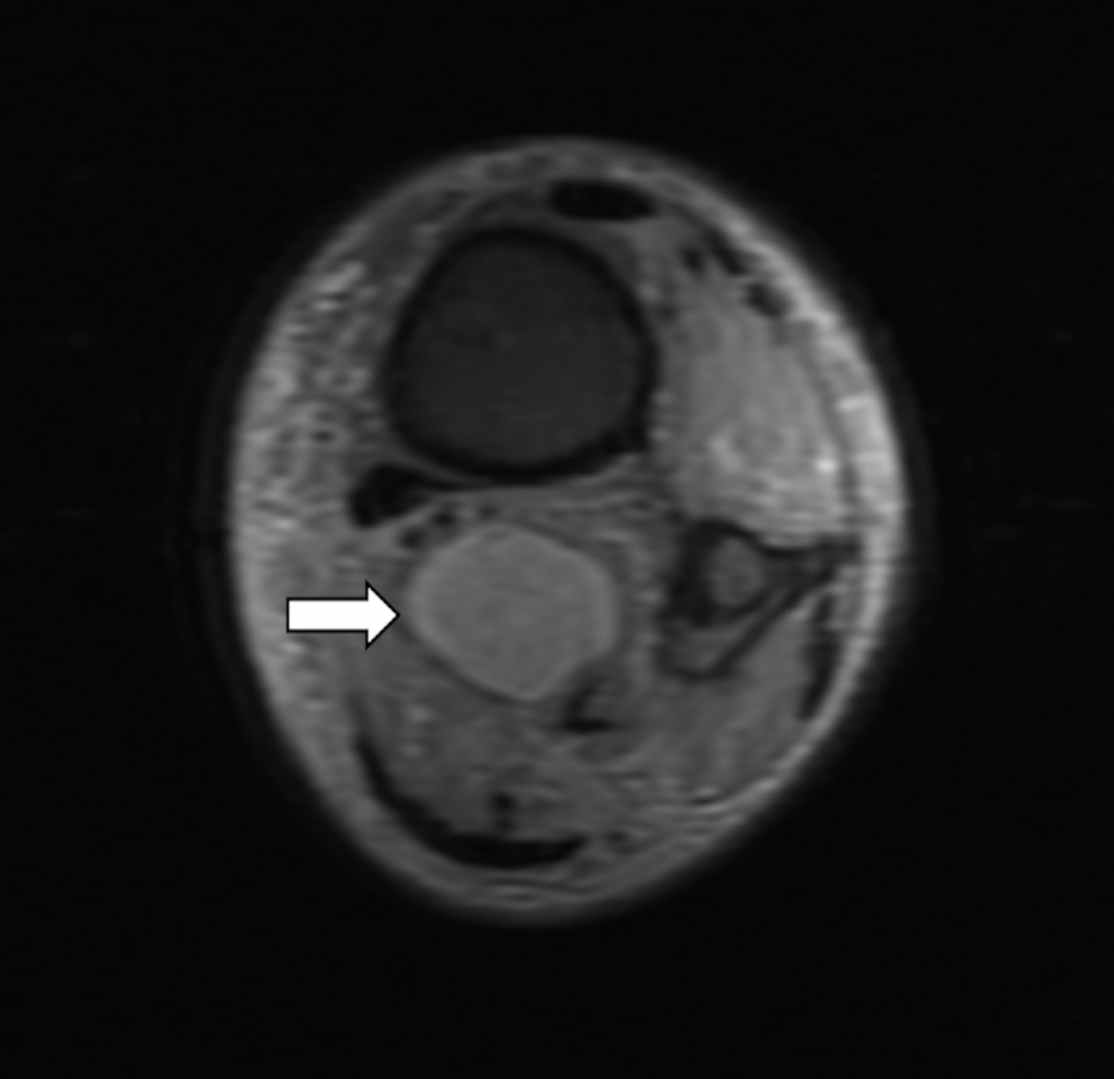
T2 weighted cross-sectional MRI showing the mass arising from posterior tibial nerve

**Figure 3:**
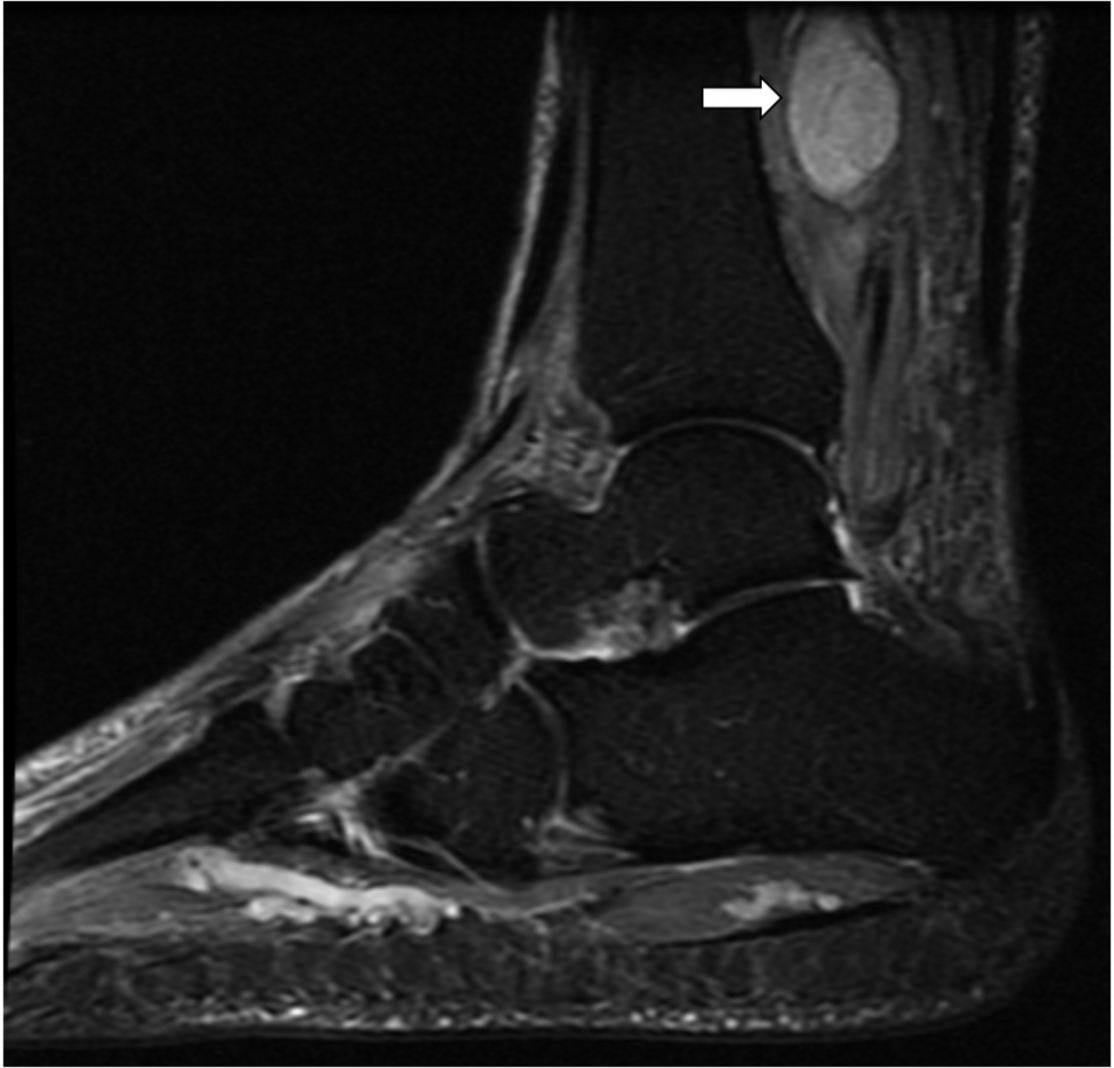
T2 weighted sagittal MRI showing the mass arising from posterior tibial nerve

The MRI of the left ankle showed a mass resembling “egg on a string” that appeared to be a peripheral nerve sheath tumor (Figures 1-3). This mass showed enhancement on T2 and post-contrast. It seemed well-encapsulated without significant edema surrounding it.

Management options were discussed with the patient and he decided to proceed with surgical removal of the mass.

**Figure 4:**
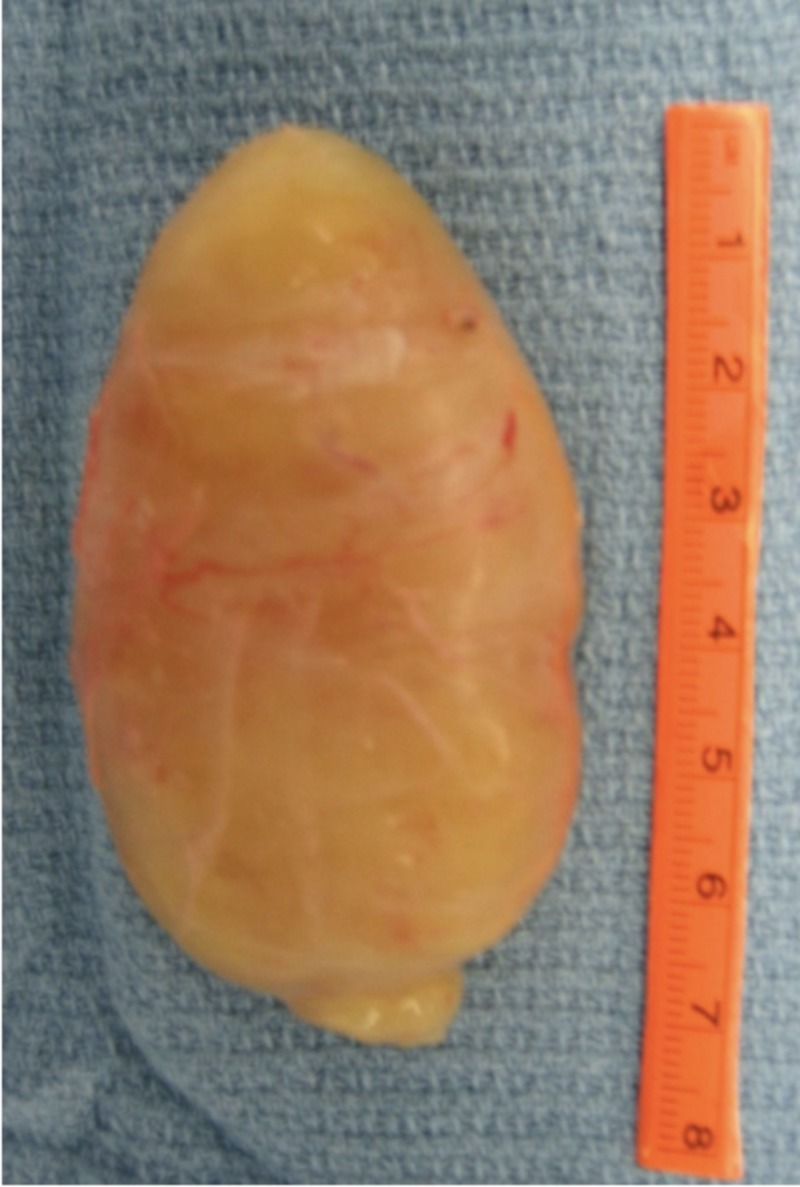
The resected tumor

**Figure 5:**
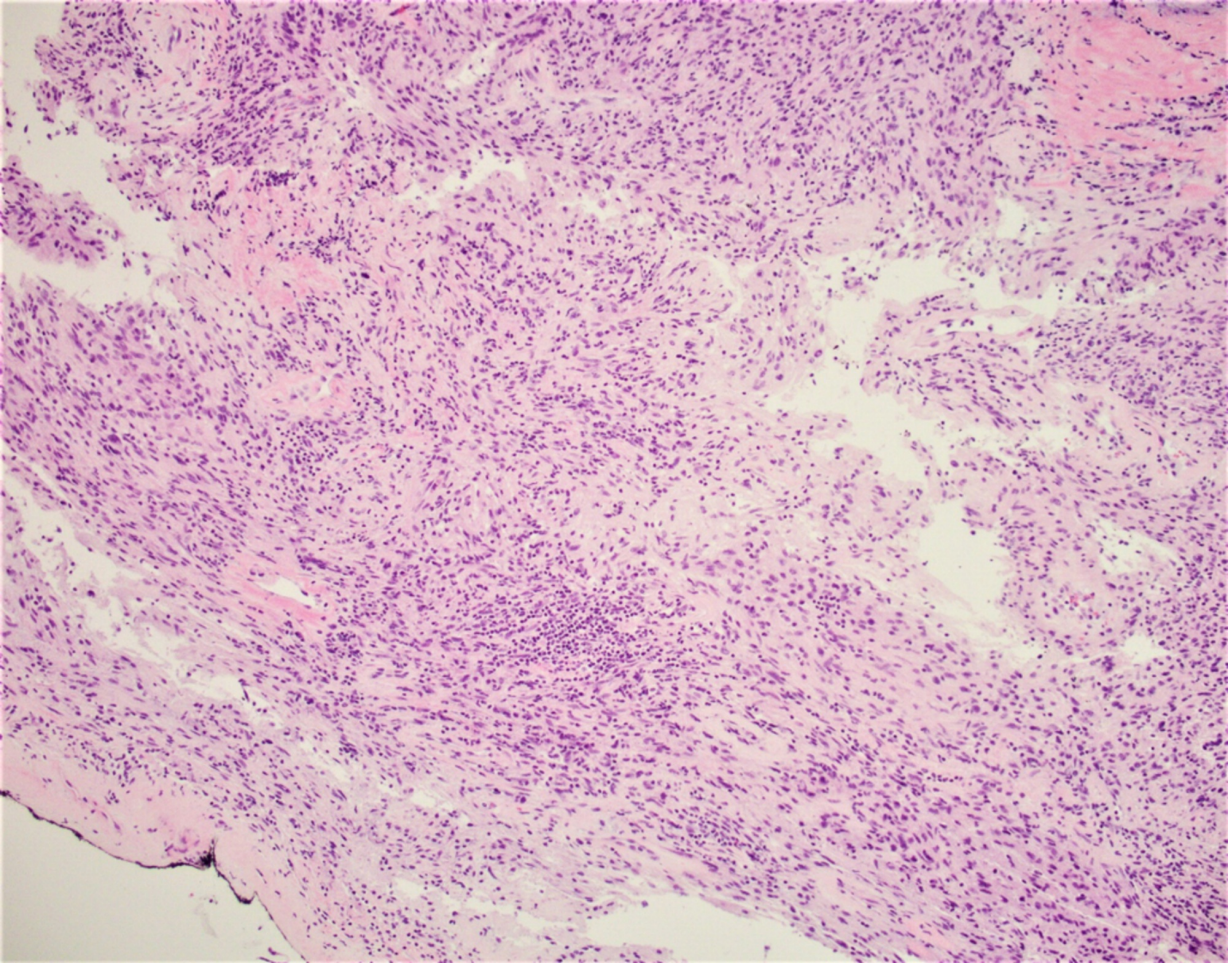
A microphotograph exhibiting the tumor presence at the inked resection margin

**Figure 6:**
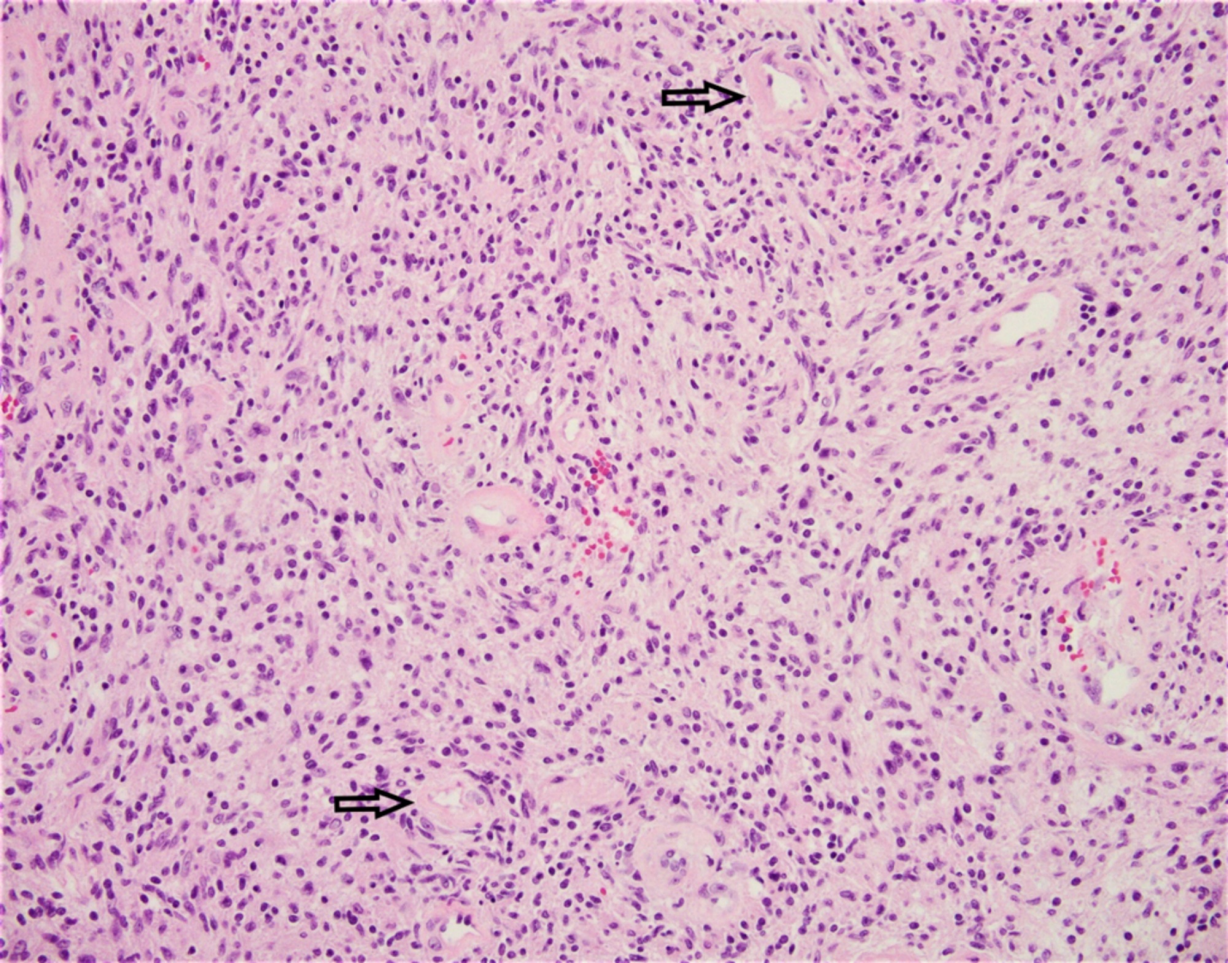
Schwannoma is a benign, usually nonrecurring nerve sheath tumor. Histologically, the tumor is composed of spindle Schwann cells with eosinophilic cytoplasm and basophilic nuclei in a collagenous stroma with thick, hyaline walls vessels [arrows].

During surgery, the tarsal tunnel was released completely. Then, the tumor was identified (3 cm long, 2 cm wide and 1.5 cm thick) to be arising from the sheath of the tibial nerve approximately 5 centimeters proximal to the tip of the medial malleolus (Figure 4). A complete tumor enucleation was carried out and the specimen was sent for a histopathological examination (Figures 5-6), which confirmed it to be a Schwannoma. The histopathology also confirmed complete enucleation. After ensuring patency of the nerve after tumor enucleation, a nerve wrap (Stryker Corp., Kalamazoo, MI, USA) was applied around the nerve.

The patient was kept in a short leg cast for two weeks non-weightbearing followed by partial weight bearing in a long CAM boot for six weeks. After this, full weight bearing was allowed. The patient presented for follow-ups at two weeks and then at nine weeks, and did not have any wound complications. He was negative for Tinel’s sign, had good ankle and subtalar range of motion with an intact sensation along posterior tibial nerve. At his last follow up two years from the surgery, he did not have pain in the foot and/or ankle regions.

## Discussion

Schwannomas are slow growing peripheral nerve tumors arising from Schwann cells of the nerve sheath [1-13]. Though schwannoma is the most common tumor of the peripheral nerve sheath, it's rate of occurrence in the lower extremities is extremely low. The lower extremities are affected in less than 10% of all cases [1-13]. The vast majority of schwannomas are benign. However, malignant schwannomas account for 5% of peripheral nerve sheath tumors [4]. Very few studies present a schwannoma affecting the posterior tibial nerve.

Schwannomas typically appear as a slow growing palpable soft-tissue masses with symptoms similar to that of compressive neuropathy. These masses grow abnormally causing compression and improper displacement of critical nerve fascicles. Patients often present with a myriad of conditions in response to the presence of schwannomas. Clinical symptoms include pain, discomfort, swelling, weakness, and paresthesia [4]. To diagnose a schwannoma, a thorough examination of the patient’s clinical history and physical examination must be performed. Positive Tinel signs and dorsiflexion-eversion are two tests that may suggest the presence of a schwannoma of the posterior tibial nerve [6,8,11]. Addtionally, nerve conduction studies may indicate a schwannoma’s existence. Radiographs are used to eliminate bone abnormalities [4]. Ultrasonography typically display a solid, sharply delineated, oval mass [4,11]. MRI may be used to define the characteristics of the tumor. The mass appears isointense compared to skeletal muscles on T1-weighted plates and eccentric relative to the involved nerve that is displaced [4]. The tumor is hyperintense on T2-weighted images. Gadolinium may be used in conjunction with MRI to improve image contrast, allowing better assessment of the lesion [4].

Diagnosing a schwannoma may often be difficult due to several reasons. First, schwannomas are uncommon in the lower extremities. Furthermore, they may be ingrained in the soft-tissue, rendering the tumor impalpable. Lastly, lumbar radiculopathy is a common misdiagnosis of neuropathic pain around the foot in the absence of a solid mass, pathology, and imaging [3, 7, 11]. Nawabi and Sinsi reported the mean time to diagnose a schwannoma to be 86.5 months in 25 cases [7]. In the study, only 3 of 25 patients were diagnosed within a year, the longest documented delay was 30 years. All the patients in the study complained of pain with 18 patients specifically complaining of pain in the sole of the foot, and the remaining seven patients complaining of pain in the calf and the ankle. Tinel’s sign was reported to be present in all the cases. The study concluded that after excluding lumbar and pelvic lesions in patients with a long-standing history of neuropathic pain in the lower limb, a benign tumor of the peripheral nerve may be sought to explain the symptoms. 

Surgical excision and decompression of the affected nerve is the most commonly employed method in treating and managing schwannomas. A longitudinal excision over the perineurium of the middle of the tumor is considered to minimize invasiveness [4,6]. The key to preventing further neurological complications is preserving the nerve fascicle during excision [6,13]. Complete excision is recommended since incomplete resection may result in recurrence with the current reported recurrence rate being less than 5% [6,13]. Following excision, histopathological assessment can be used to further confirm the diagnosis of a schwannoma. The biphasic presence of Antoni type A and Antoni type B cells comprise schwannomas and indicate its presence in histopathological analysis. Antoni type A tissue are dense and orderly arranged; in contrast, Antoni B tissue have fewer cells and disorganized areas [4,8].

The patient in this case report was diagnosed with and treated for Morton’s neuroma, tarsal tunnel syndrome and peripheral neuropathy for six years without any symptomatic relief before an MRI was performed that revealed the posterior tibial nerve mass. Surgical removal and histopathological examination of this mass confirmed it to be a Schwannoma. Surgery also resolved the patient’s symptoms.

## Conclusions

In conclusion, while rare, lower extremity schwannomas can pose a problem for patients due to their discomfort and the delay in diagnosis. Delay in diagnosis of these slow growing neoplasms can lead to further complications such as tarsal tunnel syndrome due to compression of nearby structures such as the posterior tibial nerve. It is important to recognize these tumors early in their presentation to avoid further complications and alleviate the symptoms the patients are experiencing. A high clinical suspicion along with appropriate imaging can lead to early detection and removal of these tumors.
